# Carrier Synchronous Signal Averaging for Trending Casing Crack Propagation in Planetary Gearbox

**DOI:** 10.3390/s26051663

**Published:** 2026-03-06

**Authors:** Nader Sawalhi, Wenyi Wang

**Affiliations:** Defence Science and Technology Group (DSTG), Melbourne, VIC 3207, Australia; wenyi.wang@defence.gov.au

**Keywords:** planetary gearbox casing, carrier synchronous signal averaging, crack detection, Hilbert transform, envelope analysis, health and usage monitoring systems

## Abstract

Cracks in planetary gearbox casings generate additional vibration responses, which may be used for monitoring structural degradations. This paper provides a signal processing framework to effectively track casing crack-related features in planetary gearboxes using the carrier synchronous signal average (C-SSA). The proposed algorithm is based on processing the hunting-tooth synchronous signal average (H-SSA) to extract the C-SSA which contains the cyclic interaction between the gear loadings and the corresponding casing response. The root mean square (RMS) of the C-SSA signal can then serve as a health condition indicator (CI) to track crack propagation. Further enhancement can be achieved by applying the Hilbert transform (HT) on the C-SSA using the full bandwidth to derive squared envelope signal, which enhances the trending capability. To remove cyclic temperature influences observed in the trends, singular spectrum analysis technique (SSAT) has been used to ensure that the trend reflects the changes purely due to the damage progression. Experiments using three casing-mounted sensors show good capability to track crack progression. Tests under 100%, 125%, and 150% load levels show consistent performance across these operating conditions, with better results seen at higher loads. The results demonstrate that C-SSA and its squared envelope signal effectively enhance the sensitivity and reliability of vibration-based casing crack detection, providing a practical tool for long-term structural health monitoring of planetary gearboxes.

## 1. Introduction

Planetary gearboxes are essential to helicopter main transmissions, providing high torque capacity, compactness, and load-sharing capability. Their mechanical integrity is crucial because any undetected fault can lead to significant operational and safety risks. Condition monitoring through vibration analysis has been an essential component of Health and Usage Monitoring Systems (HUMS) and Prognostic Health Management (PHM) frameworks [1]. However, vibration signals measured on planetary gearboxes are complex. They contain mixed contributions from gears, bearings and casing structures, all modulated by the carrier and planetary rotation and convolved with transfer path effects [2]. The understanding of planet gear kinematics and dynamics is very important as it explains the nature of the cyclic loading that the casing experiences. Molina [3] developed a theoretical frequency framework describing how internal and external gear meshes, together with carrier rotation, generate families of sidebands and modulation orders observable in measured vibration spectra by a sensor on the casing outside the ring gear. Molina explained that the keys to understanding vibration signals measured on planetary gearboxes are (1) phase differences between the meshing process present in the gearbox, which largely depends on the geometry of the gearbox and independent of the type of gearbox configurations. (2) phase differences between the amplitude modulation functions due to variations in the transmission paths. This depends not only on the geometry of the gearbox, but also on the measurement arrangement and/or configuration of the planets (e.g., number of planets and their spatial arrangement). Subsequent modeling work has refined the representation of these dynamics by including time-varying mesh stiffness, frictional excitation, and transfer path effects [4,5]. Together, these models establish a foundation for understanding how the cyclic loading from planetary motion drives nonlinear responses in the gearbox structure. Cyclic excitations impose repetitive stress fields on the ring gear and transmit to the casing through the ring gear and mounting interfaces.

The general body of research has focused mainly on gear and bearing faults in the planetary gear system such as spalling and pitting as these are the most common failure modes and require dedicated approaches to detect and diagnose. Structural faults in stationary components, particularly casing cracks, remain largely unexplored due to their rare occurrence and weak manifestation in measured vibration signals. The Defence Science and Technology Group (DSTG) recently conducted a controlled pinion gear spall propagation test on a decommissioned Bell 206B-1 Kiowa main rotor gearbox (Bell Textron Inc., Fort Worth, TX, USA). During this test, a casing crack developed unexpectedly and progressed to the failure of the gearbox under different load conditions (100%, 125%, and 150%). The resulting dataset captured the progression of this structural fault with high-resolution vibration data [6]. This dataset provided a unique opportunity to study the dynamic response of a real gearbox casing undergoing cyclic fatigue, an area that has previously lacked experimental validation.

The phenomenon of crack breathing, the periodic opening and closing of a fatigue crack under dynamic loads, has been studied extensively in structural dynamics and fracture mechanics. Early investigations demonstrated that breathing cracks introduce a time-varying stiffness and modulation in vibration amplitude and frequency [7,8,9,10]. Long et al. [11] advanced this concept by representing a cracked beam as a bilinear two-degree-of-freedom (2-DOF) system, where stiffness alternates with each load cycle. This lumped-parameter approach shows that breathing cracks generate sub-harmonic and super-harmonic components in the response, which vary systematically with crack size and location. Along these lines, the gearbox casing coupled with the ring gear may be intuitively treated as a 2-DOF structure in which the casing behaves as a spring with stiffness that breathes under the cyclic forces transmitted from the planetary gear set.

Recent studies on vibration-based crack fault detection have expanded the analytical and computational tools for identifying such nonlinear effects. Madisetty et al. [12] reviewed advances in vibration-based crack detection and emphasized the integration of analytical, numerical, and soft computing methods for structural health monitoring. Suresha and Reddy [13] demonstrated that the nonlinear spectral signatures of breathing cracks can be captured effectively using bilinear models and harmonic analysis. Yan et al. [14] proposed a dynamic identification method for bi-linear systems that distinguishes stiffness regions by their natural frequencies, while Qiu et al. [15] provided a finite element analysis framework linking the harmonic content of a vibrating cracked-beam to crack depth and position. These developments have significantly improved the sensitivity and interpretability of crack detection techniques and motivated the use of signal processing methods capable of isolating breathing-related nonlinearities.

Experimental and diagnostic studies demonstrated how these nonlinear features manifest themselves in vibration signals. Afolabi [16] introduced an anti-resonance-based technique that exploits sharp amplitude drops caused by destructive interference, features that are modified by crack presence and are particularly relevant in stiffened casing structures with strong modal interaction. Bovsunovsky and Surace [17] demonstrated that superharmonic components are effective for early crack detection in beam-like structures, whose nonlinear dynamic behavior is comparable to gearbox casings subjected to distributed excitation. Long et al. [11] showed that crack breathing distorts the vibration waveform and generates subharmonic, superharmonic, and intermodulation components. Non-integer harmonics such as ½× and ⅓× were identified as indicators of fatigue damage and were correlated with crack parameters, improving diagnostic relevance for practical monitoring.

Building on these understandings and foundations, the present study introduces a signal processing framework for detecting and tracking casing crack response in planetary gearboxes. The method derives a carrier synchronous signal average (C-SSA) signal from the hunting-tooth synchronous signal average (H-SSA) data to enhance features linked to the cyclic interaction between gear loading and structural response. Hilbert-based squared envelope analysis is then used to reveal modulations’ behavior and enhance trending ability, while singular spectrum analysis technique (SSAT) removes temperature-related cyclic effects to expose the true trend of damage. The framework is validated using multi-sensor vibration data from DSTG’s HUMS 2025 dataset [6] across multiple load conditions.

The paper is organized as follows: After this introduction (Section 1), Section 2 presents the materials and methods. This section describes the test and the vibration dataset and discusses the essence of the proposed signal processing algorithm. Section 3 presents the results achieved from using the proposed algorithm. Comparisons are shown between early and late stages of the crack propagation as well as trend plots using the root-mean-square (RMS) of the C-SSA and the RMS of the squared envelope signals. SSAT results are also presented in Section 3. Section 4 provides detailed discussion of the results providing physical interpretation of the crack breathing effects seen. Sensors responses under different loads and the performance of the proposed framework are also discussed in this section. Finally, Section 5 provides summary, conclusions and future work. 

## 2. Materials and Methods

### 2.1. Test Description and the Vibration Dataset

Vibration data was collected during a controlled endurance test of a helicopter main rotor gearbox, conducted by DSTG at their Helicopter Transmission Test Facility (HTTF). The gearbox consists of two reduction stages: a spiral bevel gear stage with a ratio of 3.7368:1 (71/19) and a planetary stage with a ratio of 4.6666:1 (99/27 + 1), producing an overall reduction ratio of 17.4386:1. The test article is a Bell-206B main rotor gearbox with the 4-planet (35 teeth) configuration. In the three-planet configuration, the planets are evenly distributed around the sun gear, and all mesh interactions occur in phase. This symmetry suppresses vibration orders that are not integer multiples of three. The present study uses the four-planet configuration. In this arrangement, the planets form two diametrically opposed pairs with a non-uniform angular spacing around the sun gear, approximately 88.6°, 91.4°, 88.6°, and 91.4°. Planets within opposing pairs mesh 180° out of phase. As a result, odd harmonic orders cancel between opposite planets, while even orders remain in phase and are reinforced.

The test started in October 2023 and was originally designed to monitor the progression of an artificially induced gear-tooth spalling fault in the input pinion. Testing was performed using a DC motor to drive the input shaft to the gearbox at 6000 RPM, resulting in an output speed of ~344 RPM. The gearbox was operated under a load sequence of 100% → 125% → 150% → 125% → 100% of rated torque, holding each load for ~20 min per cycle. A total of 22 days of testing was completed. The present dataset includes 86 load cycles recorded between Day 3 and Day 22, excluding Days 1–2 due to temporary control board malfunction. In May 2024, the test was terminated following the unexpected failure of the upper casing caused by a fatigue crack. Post-test fractographic analysis of the fracture surfaces suggested that the primary crack initiated at the spline locating the ring gear in the upper casing, propagated to a stud securing the casing to the support structure, and produced a secondary crack extending from the spline edge into the casing wall, resulting in a partial fracture. The ring gear remained intact, and the planetary gear set continued to operate. This casing failure does not implicate gearbox performance or reliability, as the test unit was retired from service and subjected to severe overload to accelerate gear fault progression. The publicly released HUMS 2025 Data Challenge dataset [6] differs from the dataset used in this study, where C-SSA data was included and different data records were used for all three load levels at 100%, 125% and 150% of the rated load.

**Figure 1:**
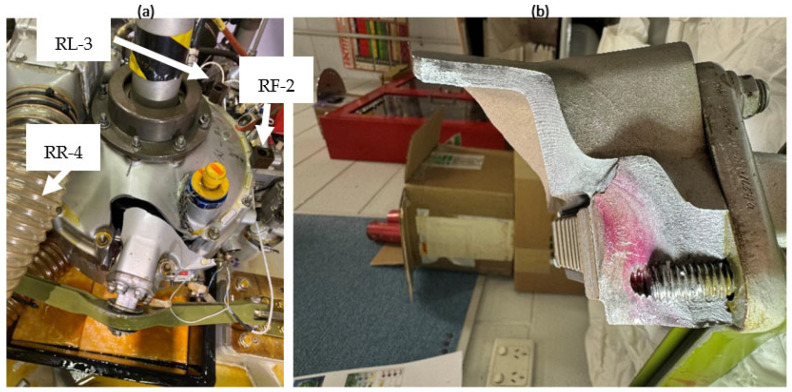
(**a**) The cracked casing of the gearbox after failure; (**b**) side view of the fracture surface of the casing.

**Figure 2:**
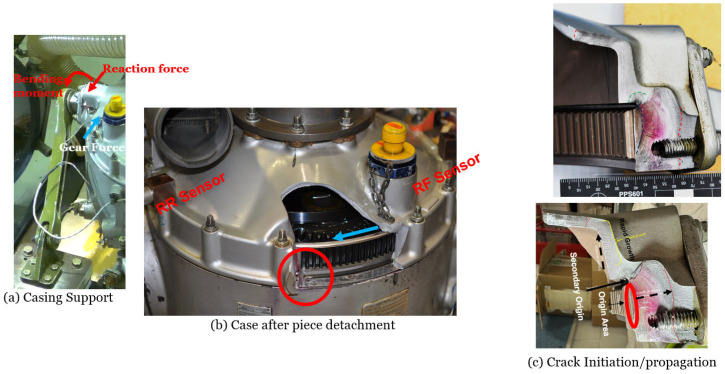
(**a**) Casing support with reaction forces; (**b**) the upper casing after piece detachment; (**c**) crack initiation and propagation.

Figure 1a shows the cracked casing of the gearbox after the failure and the locations of two casing sensors: Ring Front (RF-2) and Ring Left (RL-3). Note that the third casing sensor, Ring Rear (RR-4), is obscured by the flexible duct; however, its location has been highlighted and can be seen clearly in Figure 2. Figure 1b shows a side view of the fracture surface.

Figure 2a illustrates the structural configuration of the gearbox casing support and the applied load path. The blue arrow indicates gear forces, while red arrows represent the resulting reaction force and bending moment acting at the casing interface. Figure 2b shows the RF-2 and RR-4 sensors which are located close to the high-stress region (red circle) where the crack initiated. Oil leakage observed during the early test stages was initially attributed to a leak from the sighting glass but later identified as an early symptom of casing failure. Figure 2c shows the main crack origin area (red ellipse: C1) and its outward direction of propagation as well as the secondary origin (C2) and its upward propagation direction.

**Figure 3:**
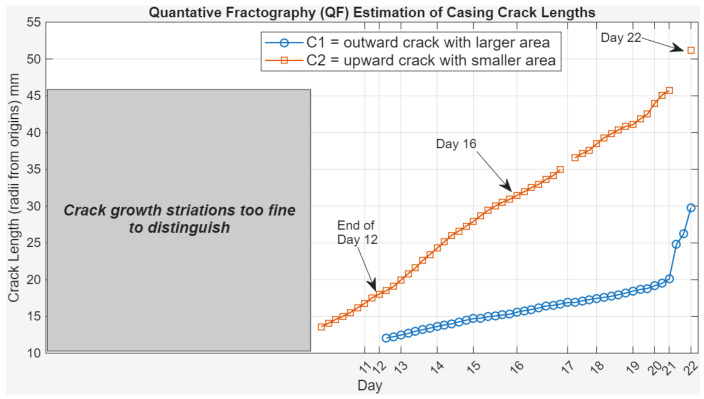
Quantitative fractography for casing crack length vs. day of testing.

Figure 3 presents a quantitative fractography analysis of the casing crack growth under cyclic loading to map the evolution of crack length over time. The figure highlights two crack fronts: C1, an outward-propagating crack with a larger crack area (shown in blue), and C2, upward-propagating crack with a smaller crack area (shown in orange). Crack length in millimeters is plotted against testing days, with overlaid key points marked at Days 12, 16 and 22. These milestones illustrate a clear and steady progression of crack growth.

Raw vibration data was pre-processed (angular resampled) with the aid of the tachometer to produce an H-SSA with 405,405 points per channel. This length was selected so that the data encompassed an integer number of revolutions of both the planet carrier and the planet gears, enabling direct derivation of either C-SSA or planet synchronous signal average (P-SSA) from the same dataset. The H-SSA was computed from raw vibration signals spanning 12 planet–ring hunting-tooth periods, with each period corresponding to 99 revolutions of the planet gears (99 × 4095 samples) or 35 revolutions of the carrier (35 × 11,583 samples). This ensured strict angular synchrony among gear, carrier, and casing signals, an essential prerequisite for coherent averaging and modulation analysis. Each H-SSA signal was standardized by subtracting its mean and dividing by the standard deviation. A total of 1723 H-SSA records were analyzed split between the three loads as follows: 434 records at 100% load, 860 records at 125% load and 429 records at 150% load.

### 2.2. Signal Processing Framework

**Figure 4:**
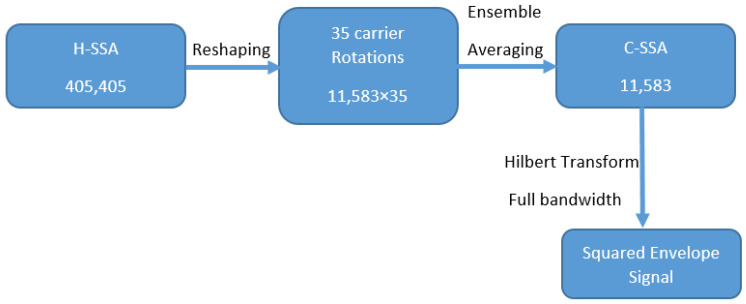
Schematic flow diagram of the proposed signal processing algorithm.

Figure 4 presents a schematic flow diagram illustrating the main processing steps proposed to extract and track casing crack-related features. The process involves reshaping the H-SSA signal (405,405) into 11,583 × 35 (35 carrier rotations with each rotation having 11,583 samples) then calculating the ensemble average of these 35 rotations. This gives a C-SSA of 11,583 samples. The RMS of each C-SSA signal can then be calculated and used as a health condition indicator (CI). The squared envelope of the C-SSA can also be obtained using a Hilbert transform applied on the full bandwidth. This helps further enhance trending capability by trending the RMS of the squared envelope signal. Note that H-SSA signal length was set to a 405,405 sample to be divisible by both 35 carrier revolutions and 99 planet gear teeth. This selection ensures simultaneous alignment with the carrier rotation and planet mesh periodicities, enabling robust extraction of the carrier-synchronous component as well as planet-synchronous component if needed while suppressing non-synchronous content.

#### 2.2.1. C-SSA and Its Squared Envelope Signal

**Figure 5:**
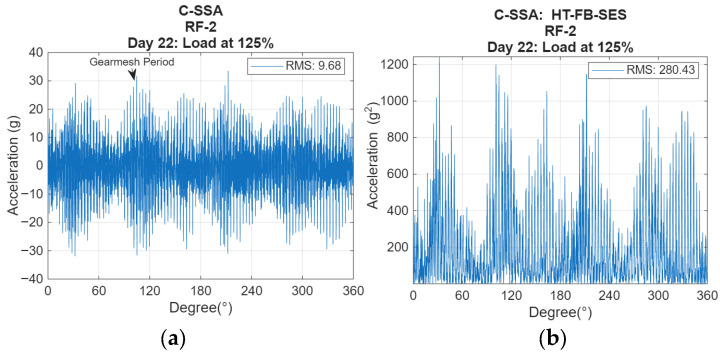
(**a**) C-SSA RF-2 for Day 22 at 125%; (**b**) C-SSA HT-FB-SES RF-2 for Day 22 at 125%.

C-SSA enhances vibration components related to the cyclic loading transmitted from the rotating carrier and planetary gear set into the flexible casing structure. Averaging across equivalent carrier cycles suppresses asynchronous gear-mesh contributions and highlights casing responses excited per carrier revolution. This transformation isolates the cyclic stiffness variation that occurs when the crack opens and closes under dynamic load, referred to as the breathing effect. Because the breathing introduces amplitude and phase modulations at carrier frequency multiples (including the planet pass frequency at 4× carrier frequency), C-SSA provides a physically meaningful representation of this interaction. Figure 5a depicts a typical C-SSA signal from RF-2 at load 125% from the last day of testing. Gear mesh periods (≈at 3.605°) and transfer path modulations are clearly seen in the signal.

To further enhance these modulations, the Hilbert transform (HT) is applied to the C-SSA over the full bandwidth (FB) to produce its analytic signal. The Hilbert approach allows precise digital demodulation and ideal band selection, overcoming the limitations of analog filtering [18]. The squared envelope signal (SES) of this analytic signal emphasizes amplitude modulations superimposed on the structural resonance bands. The evolution of these envelope features with increasing load and time provides a quantitative measure of the crack’s breathing activity. Figure 5b displays C-SSA corresponding SES using HT on the FB (HT_FB-SES) which better shows both amplitude and modulation effects.

The RMS of the C-SSA and of its HT-FB-SES are computed and used as condition indicators, capturing the global vibration energy variation over time. An increase in RMS indicates higher structural compliance and energy transmission due to crack growth as a direct result from decreased stiffness which causes increased vibration amplitudes.

#### 2.2.2. Singular Spectrum Analysis Technique (SSAT) for Trend Extraction and Temperature Correction

Vibration trends derived from RMS for both the C-SSA and its squared envelope signal were further processed using singular spectrum analysis technique (SSAT) to remove temperature variations observed in the trends. SSAT is a data-driven decomposition technique that separates a time series into interpretable components representing trend, oscillation, and noise [19,20]. It requires no a priori model and is well suited for non-stationary, nonlinear mechanical signals. SSAT involves two main stages of processing: namely decomposition and reconstruction [21]. Each of these stages has two separate steps. The decomposition stage on one side aims at separating the signal into components of trend, seasonal (cyclic) and error (random) though embedding and singular value decomposition. The reconstruction stage on the other side rebuilds the data into a new time series based on the values obtained from the decomposition step using grouping and diagonal averaging [21].

Given a discrete time series *x*(*t*) = {*x*_1_, *x*_2_, …, *x_N_*}, SSAT constructs a trajectory matrix X through Hankel embedding (one dimensional converted into multi-dimensional form) as shown in Equation (1):

(1) $$X=\left[\begin{array}{cccc} x_{1} & x_{2} & \ldots & x_{K}\\ x_{2} & x_{3} & \ldots & x_{K+1}\\ \vdots & \vdots & \ddots & \vdots \\ x_{L} & x_{L+1} & \ldots & x_{N} \end{array}\right]$$

where N: Number of samples

L: Window length (2<L<N/2) and

K=N−L+1.

Embedding stage involves the selection of a window length (*L*), where (2<L<N/2) and the data does not contain missing samples.

The second step of the decomposition stage involves the use of singular value decomposition (SVD) to separate different components. The trajectory matrix X is decomposed using singular value decomposition (SVD):

(2) $$X=U\sum V^{T}$$

where $U\in R^{L\times L}$ and $V\in R^{K\times K}$ are orthonormal matrices, and $\sum \in R^{L\times K}$ is a diagonal matrix containing singular values $\sigma_{1}\geq \sigma_{2}\geq \ldots \geq \sigma_{d}>0$, with d=rank(X).

Each singular triplet $\left(\sigma_{i},u_{i},v_{i}\right)$ defines an elementary matrix:

(3) $$X_{i}=\sigma_{i}u_{i}v_{i}^{T},i=1,2,\ldots ,d.$$

The singular values rank the contribution of each component. Large singular values typically correspond to slow-varying trends, intermediate values to cyclic or seasonal effects, and small values to noise. To reconstruct meaningful components, the elementary matrices are grouped by selecting index sets G:

(4) $$X^{\left(G\right)}=\sum_{i\in G}X_{i.}$$

In this study, the dominant components associated with the largest singular values were grouped to represent the long-term trend, while components associated with cyclic temperature effects were excluded. The final step converts the grouped matrix $X^{\left(G\right)}$ back into a one-dimensional time series $\tilde{x}(t)$ using diagonal averaging:

(5) $$\tilde{x}(t)=\frac{1}{n_{t}}\sum_{(i,j)\in D_{t}}X_{ij}^{\left(G\right)},t=1,\ldots ,N,$$

where $D_{t}$ denotes the set of matrix elements lying on the t-th anti-diagonal and $n_{t}$ is the number of elements in that diagonal. The reconstructed trend $\tilde{x}(t)$ represents a temperature-corrected, slowly varying indicator that preserves damage-related progression while suppressing cyclic environmental effects. This trend is used in subsequent analysis for crack evolution assessment and clustering-based monitoring.

The window length *L* is the only parameter to choose in the decomposition stage to achieve a better separability of a periodic component with an integer period. Hassani [21] advised for the window length (*L*) to be proportional to that period, i.e., multiple integer of the fundamental period. Furthermore, Hassani [21] indicated that it is advisable to choose an *L* reasonably large but smaller than *N*/2. Note that in the case of trending we are not interested in the cyclic components, but with the slow varying component which has the highest Eigen value. The average number of files/day (representing a number of loading cycles) is 22 for the 100% and 150% and 45 for the 125% case. As such *L* was selected to be 45 samples in the processing of trends for all loads.

**Figure 6:**
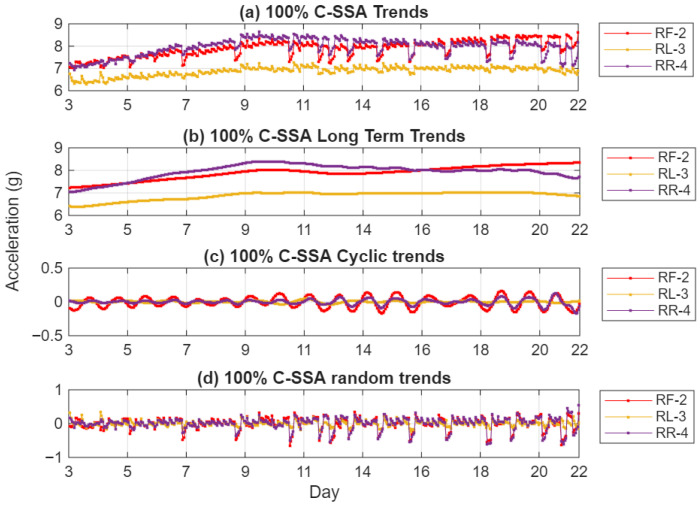
(**a**) 100% RMS C-SSA trends; (**b**) 100% RMS C-SSA long-term trends; (**c**) 100% RMS C-SSA cyclic trends; (**d**) 100% RMS C-SSA random trends.

Applying SSAT to the RMS indicators produces temperature-compensated crack-growth trends that can be more directly correlated with measured crack length and testing day. This final stage enhances interpretability and reliability of the condition monitoring process for long-term assessment. Figure 6 illustrates application of SSAT to C-SSA RMS trends for the 100% load case. The original trend (Figure 6a) shows some dips associated with the cold starts after nightly shutdowns in addition to some small cyclic trends between different measurements due to the cyclic loading variation. The long-term (LT) trend extracted and presented in Figure 6b shows a smooth clean trend after the removal of cyclic components and random components shown in Figure 6c and Figure 6d respectively.

## 3. Results

The vibration analysis algorithm proposed in Section 2.2 focuses on three fundamental techniques to track the evolution of the casing crack. First, the C-SSA is used to extract the cyclic response associated with carrier rotation. Second, HT-FB-SES extracts the amplitude modulation from the C-SSA. Third, SSAT is applied to remove environmental effects, mainly temperature-induced variations, to obtain a clean trend of structural deterioration. The results discussed in the section demonstrate the effectiveness of this combined approach.

### 3.1. Raw Vibration Signals (Accelerations) and Their RMS Trends

To address the limitations of conventional vibration monitoring and to clearly demonstrate the diagnostic improvement offered by the proposed method, a baseline comparison using raw (unprocessed) acceleration signals is included here.

**Figure 7:**
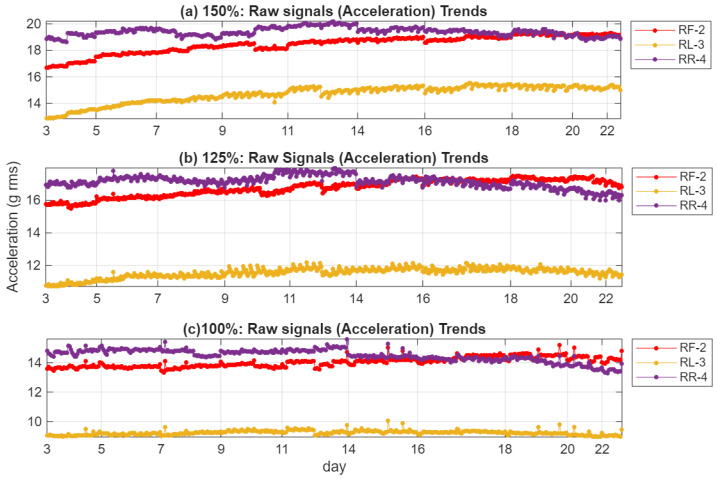
Raw acceleration signal trends (RMS or overall amplitude level in g) for the three casing sensors across different load levels. (**a**) 150% load; (**b**) 125% load; (**c**) 100% load.

Figure 7 presents the time domain trends of raw acceleration signals (expressed as overall amplitude levels or RMS in g) from sensors RF-2, RL-3, and RR-4 over the 3~22 days of monitoring period at nominal loads of 150%, 125%, and 100%. Sensor RR-4 consistently shows the highest vibration amplitudes across all load conditions. Sensor RF-2 displays intermediate levels with more noticeable day-to-day fluctuations, particularly at higher loads, indicating earlier responsiveness to structural changes near the crack initiation site. Sensor RL-3 exhibits the lowest amplitudes and the least temporal variation. While load-dependent trends are evident, the raw time-domain signals provide only limited contrast and fail to clearly separate progressive fault development from normal operational variability at an early stage.

**Figure 8:**
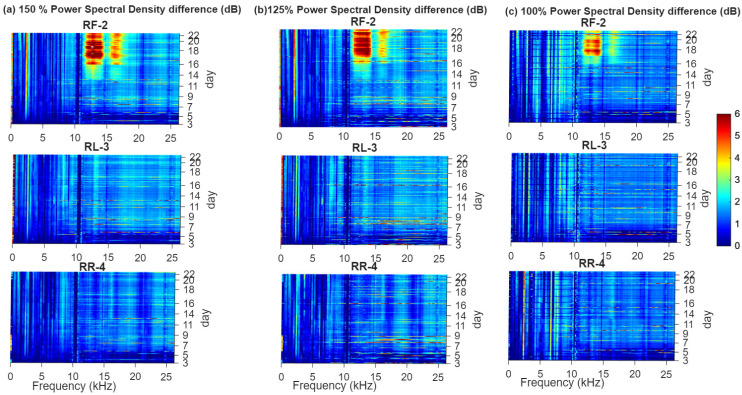
Power spectral density (PSD) difference (in dB) relative to the initial healthy state for the three casing sensors across different load levels. (**a**) 150% load; (**b**) 125% load; (**c**) 100% load.

Figure 8 complements this analysis by showing power spectral density (PSD) differences (in dB) relative to the initial healthy baseline for the same sensors and load levels, illustrating the frequency-domain evolution. Sensor RF-2 exhibits the most pronounced broadband increase in the 10–15 kHz range, which grows progressively over time and is especially prominent under 150% and 125% loads, consistent with the emergence of crack-induced impulsive events and friction-related high-frequency energy. Sensor RL-3 and sensor RR-4 show only minor, scattered PSD changes. Although these PSD maps already reveal emerging fault-related patterns more clearly than the time-domain trends in Figure 7, the signatures remain subtle and susceptible to masking by operational variability early indication and reliability for monitoring casing crack evolution.

### 3.2. C-SSA Signals and Their RMS Trends

**Figure 9:**
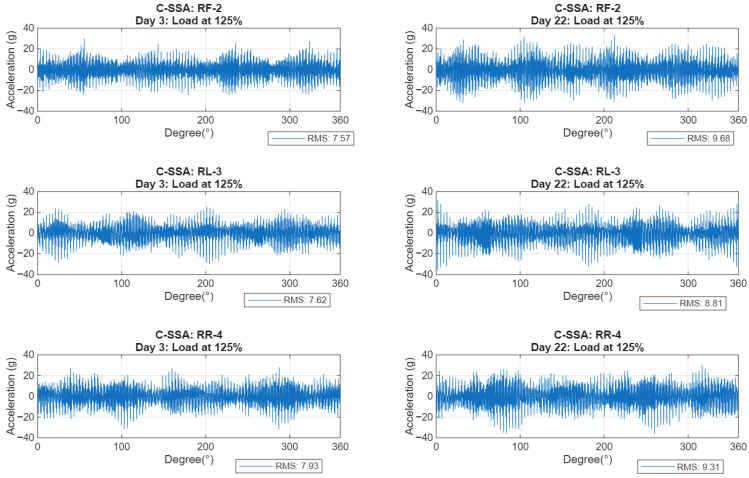
C-SSA signals at an early stage (**left**: Day 3) and late stage (**right**: Day 22) of the crack at 125% load for RF-2 (**top**), RL-3 (**middle**) and RR-4 (**bottom**).

Figure 9 presents typical C-SSA waveforms at load level of 125% comparing the early test stage (Day 3) with the advanced stage (Day 22) for the three casing sensors (RF-2, RL-3 and RR-4). This figure reveals an increasing vibration amplitude reflecting the advancement of the casing crack. The RR-4 sensor, positioned closest to the crack initiation point, records a root mean square (RMS) increase of 17.4%, while the RF-2 sensor, aligned with the direction of load reaction and maximum bending stress, exhibits a more pronounced increase of 27.9%. The RL-3 sensor, located furthest from the crack, shows the lowest increase at 15.6%.

**Figure 10:**
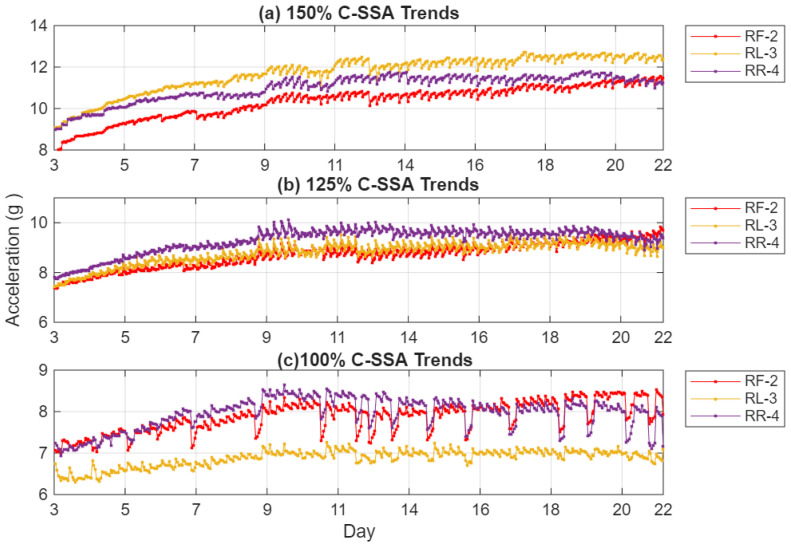
C-SSA trends at (**a**) 150%, (**b**) 125% and (**c**) 100%.

The C-SSA RMS trend, presented in Figure 10, shows a steady and almost monotonic rise in the C-SSA energy across all load cycles. This increase is directly linked to the progressive reduction in local stiffness near the crack. At 100% load, the RR-4 sensor records a root mean square (RMS) increase of 12%, while the RF-2 sensor exhibits a more pronounced increase of 19%. The RL-3 sensor shows a modest 3% increase. At 150% load, these values increase to 34.1%, 16.4%, and 24.4% for RF-2, RR-4, and RL-3, respectively. The RR-4 sensor’s proximity to the crack enables it to capture localized vibration changes effectively, while the RF-2 sensor’s alignment with the load reaction direction enhances its sensitivity to the dynamic effects of stiffness loss and cyclic deflection, resulting in the highest correlation with crack growth. In contrast, the RL-3 sensor, being furthest from the crack, shows limited response at 100% load but increased amplitude at higher torque, indicating that casing distortion extends the strain field to its location as loading intensifies. The RR-4 sensor’s response stabilizes at later stages, likely due to stress redistribution as the crack extends beyond its local region. These spatial differences confirm that the observed vibration changes are localized manifestations of structural degradation, emphasizing the critical role of sensor placement near stress concentration zones for early crack detection. Compared to the raw acceleration trends in Figure 7, the C-SSA processing substantially reduces background noise and operational variability, resulting in smoother, more consistent upward trends that better reflect progressive structural degradation.

### 3.3. C-SSA Hilbert Squared-Envelope Signals and Their RMS Trends

**Figure 11:**
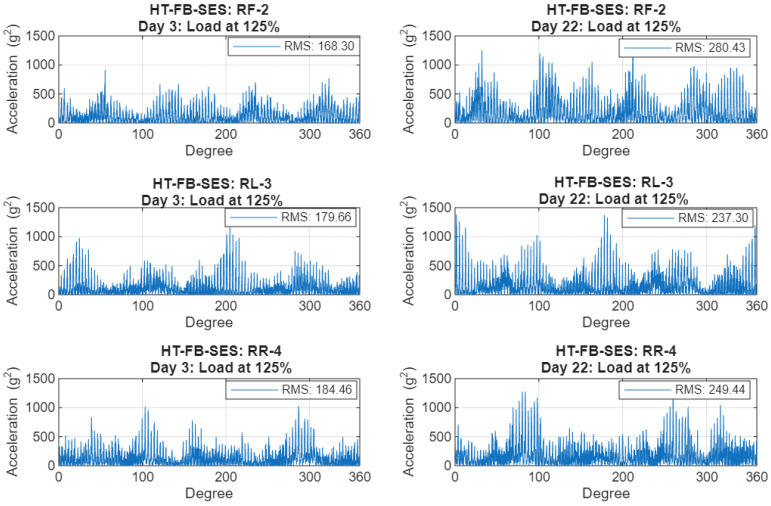
HT-FB-SES of C-SSA signals at an early stage (**left**: Day 3) and late stage (**right**: Day 22) of the crack at 125% load for RF-2 (**top**), RL-3 (**middle**) and RR-4 (**bottom**).

The HT-FB-SES effectively captures the amplitude modulation resulting from the crack’s breathing mechanism. Figure 11 illustrates a noticeable difference between the early (Day 3) and advanced stages (Day 22) of testing for the three casing sensors at load 125%, demonstrating significant increases in modulation depth. The RF-2 sensor exhibits a notable root mean square (RMS) increase of 66.7% in the squared-envelope signal, approximately 2.4 times greater than the corresponding C-SSA increase. RL-3 sensor and RR-4 sensor show smaller (32.1% and 35.2% respectively) but consistent RMS increases, indicating the localized effect of the crack’s progression.

**Figure 12:**
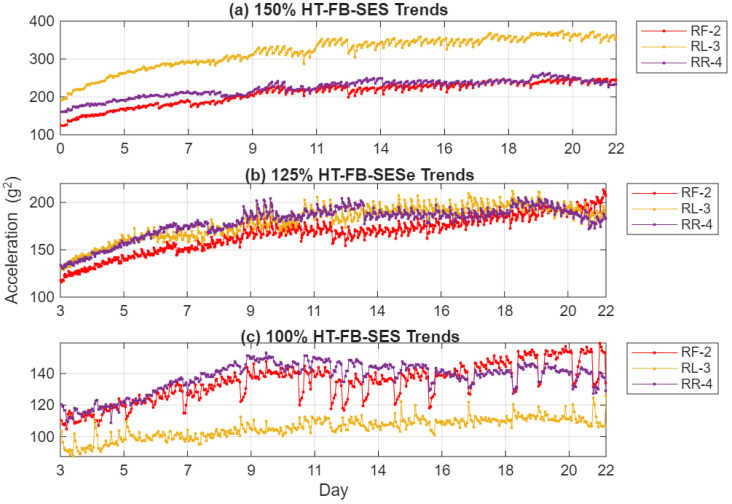
HT-FB-SES trends at (**a**) 150%, (**b**) 125% and (**c**) 100%.

The RMS evolution of the squared-envelope signal, shown in Figure 12, reveals a nearly linear and strongly monotonic upward trend across the 3~22 days of test duration for all three sensors and load conditions. Compared to the raw acceleration trends in Figure 7, the HT-FB-SES indicator provides dramatically enhanced contrast and sensitivity—typically while suppressing much of the operational variability that obscures incipient crack signatures in the unprocessed data. Relative to the C-SSA denoised trends in Figure 10, the application of Hilbert full-band demodulation further amplifies the fault-related impulsive content, resulting in clearer monotonic rises that emerge reliably by Days 5–9 even at 100% load. Sensor RF-2, aligned with the primary load reaction direction, consistently displays the highest sensitivity and the strongest correlation with crack growth across all loads. Sensor RR-4 effectively captures localized vibration changes near the crack initiation site, showing pronounced trends at higher loads, while sensor RL-3 exhibits a more limited response at 100% load but increasing activity at 125% and 150% loads, indicating that casing distortion under elevated torque extends the strain field sufficiently to influence this more distant location. Overall, these HT-FB-SES trends confirm that the combined C-SSA denoising and Hilbert full-band SES processing markedly improve early detectability, trend linearity, and diagnostic clarity compared to both raw vibration signals and intermediate denoised signals alone.

### 3.4. Temperature-Compensated Trends Using SSAT

The RMS trends derived from C-SSA and Hilbert squared-envelope analysis contain low-frequency oscillations induced by the oil outlet temperature variations. These fluctuations can mask the underlying progression of structural damage caused by the crack. To address this, SSAT was employed to isolate the mechanical trends by removing temperature-related variations while preserving the core structural response.

**Figure 13:**
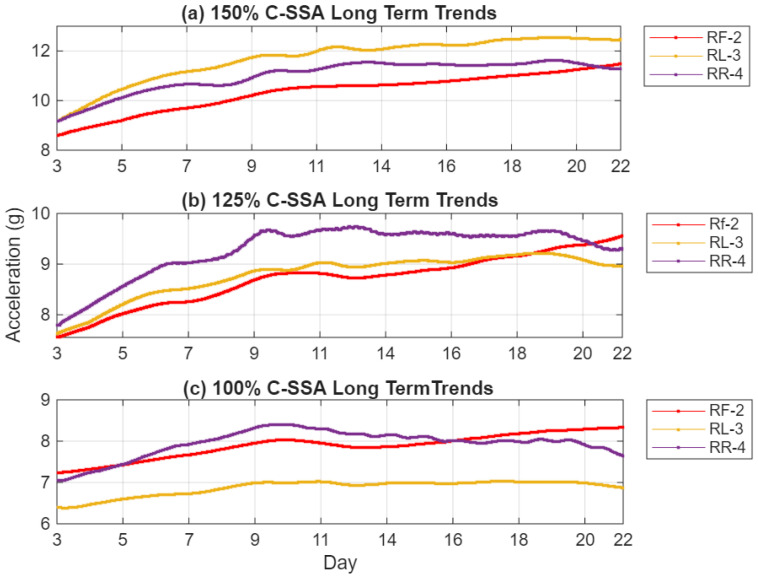
C-SSA LT trends (**a**) 150% load; (**b**) 125% load; (**c**) 100% load.

**Figure 14:**
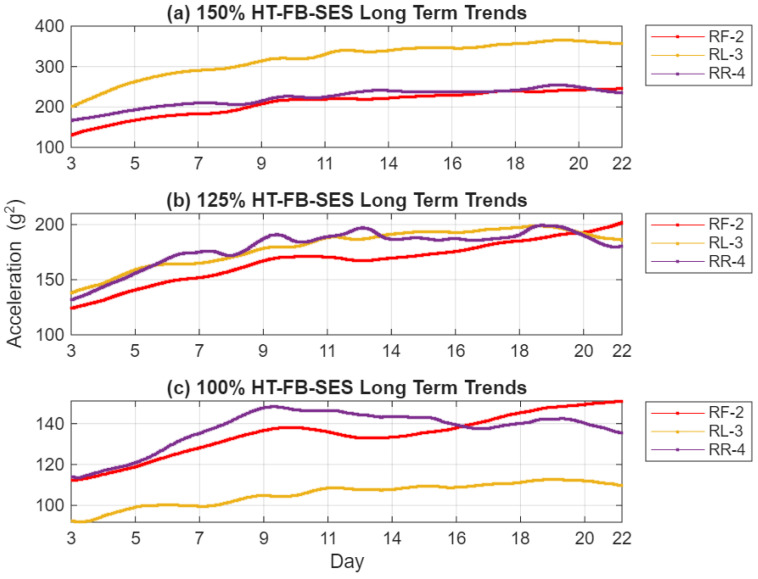
HT-FB-SES LT trends (**a**) 150% load; (**b**) 125% load; (**c**) 100% load.

Prior to SSAT filtering, the RMS trends, as shown in Figure 10 and Figure 12, exhibit some periodic oscillations that align with nightly shutdowns. After applying SSAT, presented in Figure 13 and Figure 14, temperature-induced fluctuations are effectively eliminated, revealing the smooth and monotonic increases in the RMS signals. The reconstructed trends retain only the dominant singular components associated with changes in structural stiffness, ensuring that the resulting indicators accurately reflect the progression of crack-induced deterioration.

This temperature effect correction enhances the correlation between the extracted vibration features and the measured crack length, improving the stability and reliability of the trends for long-term condition monitoring. SSAT’s ability to separate environmental effects from mechanical responses is particularly valuable for field applications, where gearbox casings are subject to continuously varying thermal and load conditions. By isolating the damage-related trends, SSAT ensures that the extracted features solely represent the physical evolution of the crack, enabling precise and consistent tracking of structural degradation over time.

### 3.5. Crack Progression Using k-Means Clustering

K-means clustering [22] partitions the indicator time series into groups by minimizing within-cluster variance while maximizing separation between clusters. In this study, k = 3 was selected to reflect three physically meaningful crack progression phases. The method operates without labeled data and requires no prior assumptions about threshold values or damage onset timing. The silhouette coefficient provides a quantitative measure of clustering quality for each day. Values close to one indicate strong agreement with the assigned cluster, while values near zero indicate overlap between clusters. Negative values suggest misclassification.

**Figure 15:**
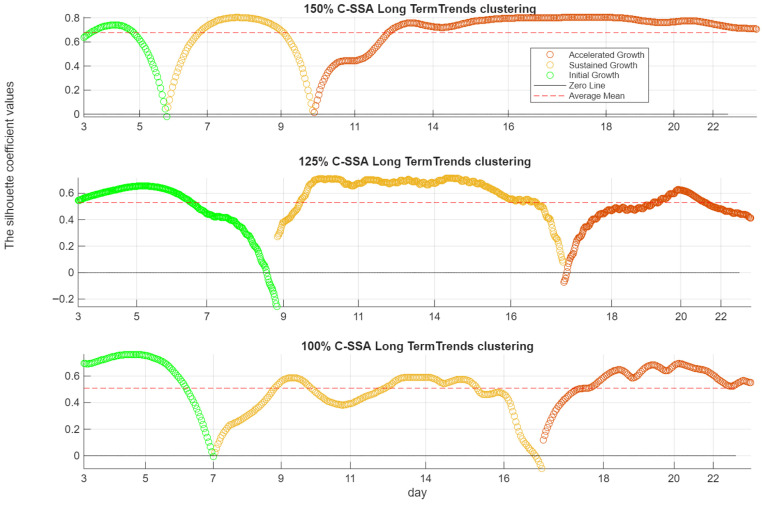
K-means clustering (k = 3) of the multivariate C-SSA denoised acceleration long-term trends (all three sensors combined) at 150% (**top**), 125% (**middle**), and 100% (**bottom**) load.

**Figure 16:**
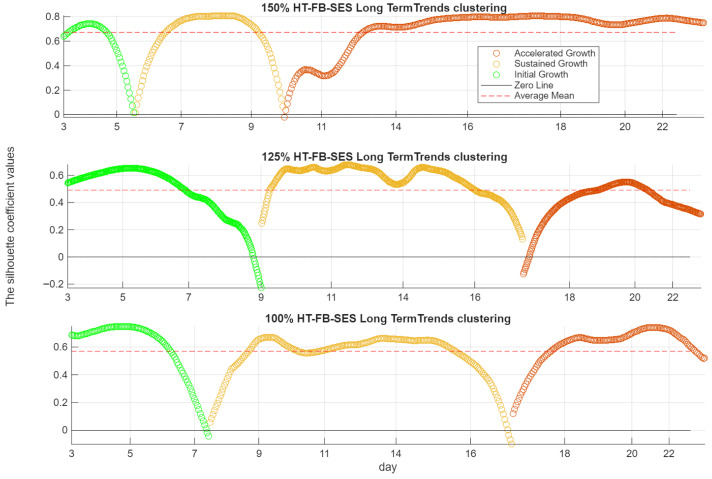
K-means clustering (k = 3) of the multivariate Hilbert full-band SES RMS long-term trends (all three sensors combined) at 150% (**top**), 125% (**middle**), and 100% (**bottom**) load.

Figure 15 and Figure 16 present the clustering results for the C-SSA and HT-FB-SES indicators, respectively, across the 100%, 125%, and 150% load cases. Each figure shows the daily cluster assignments over the 3~22-day test campaign, together with the silhouette coefficient, which quantifies the quality of the clustering for each day. Across all load cases, Figure 15 and Figure 16 show consistent clustering behavior between the C-SSA and HT-FB-SES indicators.

At 150% load, the crack evolution is clearly segmented. Initial Growth is identified from Day 3 to Day 6. Sustained Growth follows between Days 6 and 10. Accelerated Growth begins after Day 10 and persists for the remainder of the test. All silhouette coefficients remain above zero throughout this period, indicating strong cluster separation and reliable phase identification. This transition aligns well with the PSD analysis of sensor RF-2, which shows a marked increase in broadband energy within the 10–15 kHz band after Day 10.

At 125% load, the clustering remains effective but less distinct. The silhouette coefficient drops below zero around the mid-test period, indicating partial overlap between growth phases and some misclassification. Accelerated Growth is detected around Day 17. This delayed transition is consistent with the PSD response of RF-2, where increased broadband energy becomes evident around Day 14. The discrepancy reflects a more gradual damage progression compared to the 150% load case.

At 100% load, initial deviation from baseline is observed around Day 7. Accelerated Growth is identified shortly after Day 16. Despite the lower load, the indicators capture cumulative damage effects over time. The clustering reflects long-term crack evolution rather than instantaneous load magnitude alone.

## 4. Discussion

### 4.1. Physical Interpretation of Crack Breathing Behavior

Results provide strong evidence of the physical mechanism governing a breathing fatigue crack in a cyclically loaded gearbox casing. As the crack propagates, local stiffness decreases, leading to increased casing deflection and increased vibration amplitude responses. During the crack’s opening phase, the decreased stiffness amplifies vibration energy, while partial contact during the closed phase introduces nonlinear stiffness variations. This dynamic behavior increases amplitudes at each gear mesh and planet passage (modulation) and can be clearly observable in both the C-SSA and the HT-FB-SES. The progressive rise in root mean square (RMS) energy reflects the combined effects of stiffness degradation, nonlinear contact, and cyclic strain redistribution. At higher load or torque levels, prolonged crack opening intensifies the amplitude modulation, resulting in steeper RMS growth in both C-SSA and its squared envelope signal.

### 4.2. Sensor Behavior and Structural Dynamics

The spatial variation in sensor responses offers valuable insights into the structural dynamics of the casing. The RR-4 sensor effectively captured localized vibration changes being the closest to the crack location, while the RF-2 sensor exhibits the highest sensitivity and strongest correlation with crack growth. The RL-3 sensor shows limited response at 100% load but increased amplitude at higher torque, indicating that casing distortion extends the strain field to more distant regions as loading intensifies. The RR-4 sensor’s response stabilizes in later stages, likely due to stress redistribution as the crack extends beyond its local region. These distinct patterns confirm that the detected vibration changes are localized manifestations of structural degradation rather than global excitation effects. The findings highlight the compliance of the casing as a dynamic structure, transitioning from localized crack breathing to broader flexibility as damage progresses. Practically, it is often not feasible to place multiple sensors on a single component. Most helicopter HUMS would have only one sensor on the casing. Therefore, multiple sensors would help to understand the physics behind a failure mode and perhaps provide insights into how to strategically place the accelerometer to capture both local and distributed responses for effective structural diagnostics.

### 4.3. Quantitative Comparison and Sensitivity Analysis

Table 1 summarizes the root mean square (RMS) increases for the RF-2, RL-3 and RR-4, sensors across load conditions, comparing the C-SSA and Hilbert-based squared-envelope (HT-FBSES) methods. The table demonstrates a consistent increase in both time-domain (C-SSA) and HT-FB-SES RMS metrics (or CI) across all sensors, reflecting the proportional relationship between vibration energy, crack severity, and applied load. The RF-2 sensor exhibits the highest RMS increases, with values of 19.1%, 27.9%, and 34.1% for C-SSA, and 44.6%, 66.7%, and 74.7% for HT-FB-SES signals at 100%, 125%, and 150% loads, respectively, highlighting its superior sensitivity to crack-induced dynamic effects. The RR-4 sensor shows significant RMS increases (11.7%, 17.4%, and 16.4% for C-SSA; 20.0%, 35.3%, and 34.4% for HT-FB-SES), capturing localized vibration changes effectively. The RL-3 sensor, furthest from the crack, records the smallest increases (2.6%, 15.6%, and 24.4% for C-SSA; 4.4%, 32.1%, and 51.7% for HT-FB-SES), with a more pronounced response at higher loads, indicating the spread of the strain field to distant regions as the crack progresses.

The HT-FB-SES/C-SSA sensitivity ratio, ranging from 1.7 to 2.4 across the sensors and load levels, underscores the enhanced diagnostic capability of the squared-envelope method, particularly for the RF-2 sensor, which consistently shows the highest sensitivity. This ratio reflects the nonlinearity introduced by the crack’s breathing behavior and the influence of load on stiffness modulation, confirming the reliability of these metrics for early-stage crack detection and monitoring across diverse sensor placements.

### 4.4. Integrated Framework Performance

The integrated approach combining C-SSA, Hilbert squared-envelope demodulation, and SSAT filtering establishes a robust framework for detecting and monitoring breathing-related features in gearbox casings. These techniques produce consistent and interpretable indicators that align closely with physical observations and measured crack length. This methodology bridges the gap between controlled laboratory studies and real-world operational monitoring, offering a reliable foundation for gearbox diagnostics in variable thermal and load conditions. The framework’s ability to isolate and trend damage-related features enhances its applicability for continuous structural health monitoring in practical settings.

## 5. Summary, Conclusions and Future Work

This study developed and validated a robust signal processing framework for detecting and monitoring breathing cracks in planetary gearbox casings using vibration sensors mounted on the casing.

The Carrier-Synchronous Signal Average (C-SSA) effectively isolates the carrier-related vibration response. It reveals a consistent increase in root mean square (RMS) energy, directly correlating with stiffness degradation due to crack propagation.

The Hilbert-Based Squared-Envelope Analysis (HB-SES) method significantly enhances sensitivity to nonlinear amplitude modulation caused by the crack’s breathing behavior. It provides earlier and more pronounced detection of structural damage compared to traditional RMS metrics, with sensitivity ratios of 1.7–2.4 times higher across load conditions.

The inclusion of baseline comparisons using RMS trends and PSD analysis reinforces the effectiveness of the proposed C-SSA and Hilbert full-band SES indicators. The processed signals clearly enhance the detectability of localized vibration changes associated with casing cracks, particularly in sensors optimally placed relative to the crack. These results demonstrate that the methodology improves the sensitivity and interpretability of crack detection compared to raw or order-tracked vibration alone.

The Singular Spectrum Analysis Technique (SSAT) successfully eliminates low-frequency oscillations induced by environmental temperature variations, ensuring that the extracted vibration indicators accurately reflect mechanical deterioration rather than external influences. In terms of the effects of sensor placement, the RR-4 sensor, positioned closest to the crack initiation point, effectively captures localized vibration changes. Meanwhile, the RF-2 sensor, aligned with the load reaction direction and maximum bending stress, exhibits the highest sensitivity and strongest correlation with crack growth. The RL-3 sensor, located furthest from the crack, detects secondary deformation effects, particularly at higher torque levels, highlighting the spread of the strain field.

The combination of C-SSA, Hilbert squared-envelope analysis, and SSAT filtering provides a quantitative and physically interpretable approach for tracking crack progression under cyclic torque. K-means clustering demonstrates a practical and automated strategy for crack detection and monitoring. The proposed indicators identify early deviations at all load levels and consistently capture the transition to accelerated damage. Higher loads result in earlier and more clearly separated crack phases, while lower loads reveal slower but cumulative damage progression. Agreement with PSD observations confirms that the detected transitions correspond to physical changes in gearbox dynamics. These results support the use of C-SSA and HT-FB-SES as reliable indicators for condition monitoring in operational transmission systems.

This framework delivers reliable indicators that align well with observed crack growth and structural dynamics. These results demonstrate that the proposed vibration-based framework, integrating C-SSA, Hilbert demodulation, and SSAT, effectively captures the dynamic signature of breathing cracks in gearbox casings. It establishes a foundation for long-term health and usage monitoring systems (HUMS), enabling early fault detection and supporting predictive maintenance in aerospace and industrial applications.

Future research will focus on incorporating model-based feature extraction, multi-sensor data fusion, and validation to enhance the framework’s applicability for real-world deployment.

## Figures and Tables

**Table 1:** C-SSA and HT-FB-SES RMS increases for casing sensors across load conditions.

Load (%)	Sensor	C-SSA RMS Increase (%)	HT-FB-SES RMS Increase (%)	RMS Ratio (HT-FB-SES/C-SSA)
100	RF-2	19.1	44.6	2.3×
	RL-3	2.6	4.4	1.7×
	RR-4	11.7	20.0	1.7×
125	RF-2	27.9	66.7	2.4×
	RL-3	15.6	32.1	2.1×
	RR-4	17.4	35.3	2.0×
150	RF-2	34.1	74.7	2.2×
	RL-3	24.4	51.7	2.1×
	RR-4	16.4	34.4	2.1×

## Data Availability

The data used in this work was similar to that provided in a data challenge organized at the DSTG International HUMS conference [6]. The details of the test can be accessed using the following link: https://www.dst.defence.gov.au/our-technologies/hums2025-data-challenge (accessed on 17 February 2026). Data used in this paper can be made available upon request.

## Abbreviations

The following abbreviations are used in this manuscript:

H-SSA	Hunting-tooth Synchronous Signal Average
C-SSA	Carrier Synchronous Signal Average
HT	Hilbert Transform
FB	Full Bandwidth
SES	Squared Envelope Signal
SSAT	Singular Spectrum Analysis Technique
RMS	Root Mean Squared
HUMS	Health Usage and Monitoring System
PHM	Prognostic Health Management
RF-2	Ring Front Sensor 2
RL-3	Ring Left Sensor 3
RR-4	Ring Rear Sensor 4
DSTG	Defence Science and Technology Group
HTTF	Helicopter Transmission Test Facility
